# Comparative genomic analysis reveals distinct population structure in *Legionella anisa*

**DOI:** 10.1093/femsle/fnag100

**Published:** 2026-09-03

**Authors:** Maria Najeeb, Sara Matthews, Michèle Prévost, Sebastien P Faucher

**Affiliations:** Department of Natural Resource Sciences, McGill University, Sainte-Anne-de-Bellevue, Quebec H9X 3V9, Canada; Department of Natural Resource Sciences, McGill University, Sainte-Anne-de-Bellevue, Quebec H9X 3V9, Canada; Department of Civil, Geological, and Mining Engineering, Polytechnique de Montreal, Montreal, Quebec H3C 3A7, Canada; Industrial Chair on Drinking Water, Department of Civil, Geological, and Mining Engineering, Polytechnique de Montreal, Montreal, Quebec H3C 3A7, Canada; Department of Natural Resource Sciences, McGill University, Sainte-Anne-de-Bellevue, Quebec H9X 3V9, Canada; Centre de Recherche en Infectiologie Porcine et Avicole (CRIPA), Université de Montréal, Faculté de Médecine Vétérinaire, Saint-Hyacinthe, Québec J2S 2M2, Canada; Centreau – Centre québécois de recherche sur la gestion de l’eau, Université Laval, Québec G1V 0A6, Canada

**Keywords:** *Legionella anisa*, whole genome sequencing, SNP-based phylogeny, pangenome, amoeba, comparative genomics

## Abstract

*Legionella anisa* has been frequently isolated from engineered water systems; however, its population structure remains understudied compared to *Legionella pneumophila*. Here, we generated complete genome sequences for four *L. anisa* isolates recovered from a healthcare facility in Rimouski, Canada. Further the population structure of this species was investigated by performing comparative genomic analyses of the genomes generated in this study together with publicly available *L. anisa* genomes. Genome-wide phylogenetic analysis revealed the presence of three distinct clades separated by substantial genetic divergence (∼500 single nucleotide polymorphisms), with the Rimouski isolates forming a tightly clustered group, suggesting a clonal lineage. Comparative pangenome analysis indicated moderate core genome conservation accompanied by a highly variable accessory genome (∼50%). The isolates characterized in this study harbored multiple plasmids encoding genes associated with conjugation, heavy metal resistance, and other stress-related functions, suggesting potential roles in environmental persistence. Previous studies have shown that *L. anisa* can proliferate within protozoan host cells, although outcomes vary depending on the host species. Our isolates showed efficient proliferation within *Acanthamoeba castellanii*, but not within *Vermamoeba vermiformis*, under the conditions tested. Together, these findings underscore the genomic diversity of this understudied *Legionella* species and provide a framework for future investigations regarding environmental persistence and potential pathogenicity.

## Teaser

The study advances understanding of *Legionella anisa* diversity and provides a foundation for future research on its environmental persistence and disease-causing potential.

## Introduction

*Legionella* species are water-borne pathogens inhabiting natural and engineered water systems (EWS) (Steinert et al. 2007). Within water systems, *Legionella* spp. amplify intracellularly within protozoan host cells, such as amoebae and ciliates (Steinert et al. 2007). The bacteria enter human lungs through the aerosolization of contaminated water droplets (Isberg et al. 2009). It then infects alveolar macrophages and proliferates intracellularly using the Icm/Dot Type IVB secretion system to translocate effector proteins into host cells (Mondino et al. 2020). Human infection with *Legionella* can result in two clinical outcomes: Pontiac fever, a self-limiting flu-like illness, or Legionnaires’ disease (LD), a severe and potentially fatal form of pneumonia (Centers for Disease Control and Prevention (CDC) 2022). The genus *Legionella* includes 76 species, of which approximately half have been reported to cause disease in humans (LeCo Project 2024). Although the genus comprises numerous species, *Legionella pneumophila* accounts for most LD cases (∼93%–97%), with serogroup 1 representing the predominant subtype (∼84%) (European Centre for Disease Prevention and Control (ECDC) 2026). In contrast, non-pneumophila species contribute to a small proportion of reported infections (<3%) (ECDC 2026).

*Legionella anisa* is one of the most frequently detected non-pneumophila species in EWS, particularly in drinking water distribution systems and cooling towers (Crook et al. 2024, Gorman et al. 1985). Ecologically, accumulating evidence suggests that *L. anisa* occupies niches distinct from *L. pneumophila*, particularly in EWS. For instance, *L. anisa* is frequently associated with cold-water systems, whereas *L. pneumophila* seems to favor systems with elevated temperatures (De Giglio et al. 2023).

Although *L. anisa* is commonly found in environmental samples, its contribution to LD is consistently low across surveillance studies. For instance, only four LD cases have been attributed to *L. anisa* in Europe between 2019 and 2021 (ECDC 2021, 2022, 2023) and three cases in the USA between 2018 and 2019 (CDC 2023). This disparity between environmental prevalence and clinical incidence is likely explained, at least in part, by the lower virulence of *L. anisa* for humans and the lack of systematic testing for it in clinical samples, with disease occurring primarily in highly vulnerable immunocompromised populations (Crook et al. 2024).

While *L. anisa* may not represent a major concern in low-risk settings, it remains of particular importance in healthcare facilities (HCFs), where vulnerable populations are at increased risk of infection (Sylvestre and Oesterholt 2026). In such environments, identifying contamination sources through integrated approaches combining environmental sampling and genomic characterization can be valuable, as genomic diversity provides a framework to interpret strain relatedness and support source attribution. However, the genomic diversity and population structure of *L. anisa* remain poorly characterized, particularly in the context of broader genomic datasets, and the contribution of evolutionary processes such as recombination and mobile genetic elements is not well understood. In this study, we generated complete genome assemblies for four environmental *L. anisa* isolates from Canada and investigated their population structure in relation to publicly available genomes to characterize their genomic diversity. In addition, the ability of these isolates to replicate within two protozoan hosts was evaluated.

## Methods

### Sampling and culture conditions

The four new *L. anisa* isolates were obtained during an epidemiological investigation of a health care facility in Rimouski, Quebec. Water was sampled from 16 points in the HCF including from the water distribution system (i.e. hot water reservoirs and recirculation loops) and points of use such as showers, baths, faucets, and ice machines. One liter was set aside and processed as follows to isolate minor non-*pneumophila* strains based on a modified CDC protocol (CDC 2025). Cells were concentrated by filtering environmental water through a 0.45 µm filter with an additional rinse of 100 ml of sterile distilled water and then resuspended from the filter in 2.5 ml of sterile distilled water. A 200 µl aliquot of the concentrated suspension was plated directly on one ACES-buffered charcoal yeast extract (CYE) agar plates (Sigma-Aldrich), one CYE plate with 1% bovine serum albumin (BSA), one CYE plate supplemented with *Legionella* Selective Supplement (Millipore Sigma) as per manufacturer instructions (GVPC), and one CYE plate supplemented with 1%BSA and GVPC. Another 1 ml aliquot was subjected to an acid treatment, 1 ml of 0.01 M HCl/0.18 M KCl, for 5 min at room temperature before plating on each type of plate. The last 1 ml of concentrate was enriched for *Legionella* by incubation with 1 ml of *Tetrahymena pyriformis* at 5 × 10^5^ cells/ml in sterile distilledwater for 72 h at 37⁰C before plating 200 µl directly or after an acid treatment on all four plate types. Negative controls included blank plates and 200 µl of sterile distilledthat had undergone each possible condition. A positive control sterile distilled water spike with *L. pneumophila* Philidelphia-1 was used as a positive control. All plates were incubated at 37°C for 3–14 days and checked daily for colonies. Potential *Legionella* candidates were streaked onto CYE with and without cysteine. Those that could not grow in the absence of cysteine were stored as glycerol stocks (20% glycerol in AYE) and then subjected to further testing to confirm their identity. This included the *Legionella* Latex Test (Thermo Scientific) performed as per manufacturer instructions and sanger sequencing of the 16S region amplified with 27F and 1492R primers (Paranjape et al. 2022). Sequences with >99% identity in an NCBI BLAST search were attributed to that species.

### Whole genomes sequencing

Genomic DNA (gDNA) was extracted from *L. anisa* cultures grown in liquid AYE using the Wizard Genomic DNA purification kit (Promega) following the manufacturer’s instructions. DNA quantity was assessed using the Quant-iT™ PicoGreen™ dsDNA Reagent and Kit, and DNA integrity was analysed via gel electrophoresis. Short-read and long-read sequencing was performed using Illumina MiSeq and Oxford Nanopore (ONT) platforms, respectively. For Illumina sequencing, DNA was sequenced using MiSeq PE300 chemistry. Libraries were prepared by the McGill Genome Center. For ONT sequencing, High molecular weight DNA libraries were prepared by using the Rapid Barcoding Kit V14 (SQK-RBK114.96) as per manufacture instructions. Samples were sequenced on R10.4.1(FLO-MIN114) MinION flowcell.

### Genome assembly and annotation

Illumina paired-end reads were quality checked using FastQC (Andrews 2010). Adapters and low-quality reads (Phred <25) were filtered out using fastp with default parameters (Chen 2023). For ONT sequencing, sequencing, and data acquisition was carried out by the MinKNOW software. After sequencing and data acquisition reads were base called with dorado in super-high accuracy mode with configuration file dna_r10.4.1_e8.2_400bps_hac@v5.0.0 and default demultiplexing parameters were applied. Basic quality metrics and statistics of the long reads were checked using Nanoplot before downstream analyses (De Coster and Rademakers 2023).

Hybrid assemblies were generated by combining short and long reads, with Illumina short reads scaffolded on ONT long reads using NanoForms (Czmil et al. 2022). The quality of the assemblies was assessed using Bandage (Wick et al. 2015) and MiGA (Rodriguez-R et al. 2018) to evaluates % completeness, % contamination, and confirming the taxonomic assignment. Prokka was used for genome annotation (Seemann 2014). For all analysis tools, default parameters were used unless otherwise specified.

### Phylogenetic analysis and recombination detection

To assess the phylogenetic relationships between genomes from our study and previously sequenced genomes, 76 publicly available genomes (draft or complete) of *L. anisa* were downloaded from NCBI (Table S1); however, during the Gubbins analysis (see below), three genomes were automatically excluded by the software because they exhibited ∼40% divergence from all other *L. anisa* genomes and formed a distinct group that did not cluster with the rest of the dataset; therefore, they were not included in the further recombination or phylogenetic analyses.

kSNP4 was used to identify all single nucleotides polymorphisms (SNP) accross the genomes (Gardner and Hall 2013). For recombination detection and phylogenetic analysis solely based on vertically acquired point mutations, the genomes were analysed using Gubbins (Croucher et al. 2015). Briefly, assembled genome sequences of the isolates were aligned to the complete reference genome using SKA2 available with Gubbins package. Here, we selected *L. anisa* isolate UMCG_3A (2017) as the reference, since this is the oldest isolate for which a complete chromosome-level genome and geographic information is available. Further, Gubbins analysis was performed as described previously (Najeeb et al. 2025). Pairwise SNP difference between all genomes was calculated using snp-dists (https://github.com/tseemann/snp-dists).

### Comparative pangenome analysis

Pangenome analysis was conducted on the annotated genomes using panaroo (Tonkin-Hill et al. 2020). The analysis was run using sensitive mode to keep plasmids and other mobile genetic elements under consideration. Further, gene_presence_abasence.csv file generated by panaroo was used to identify the core and accessory genes in the genomes.

### Detection of antimicrobial resistance and virulence genes

All genomes were screened for antimicrobial resistance (AMR) virulence genes ABricate (Seemann 2022). Antimicrobial resistant (AMR) genes were identified against the Comprehensive Antibiotic Resistance Database (CARD) (Alcock et al. 2023) and virulence genes were screened against the Virulence Factor Database (VFDB) applying the cut-off value of 60% identity and 60% coverage.

### Intracellular growth in amoebae

Two amoeba host cells, *Acanthamoeba castellanii* and *Vermamoeba vermiformis*, were used to assess the infectivity of *L. anisa* strains included in this study. *Acanthamoeba castellanii* and *V. vermiformis* cells were routinely cultured in 75 ml vented tissue culture flasks (Sarstedt, Corning) at room temperature and passaged twice weekly in peptone–yeast–glucose medium and modified PYNFH medium supplemented with fetal bovine serum (FBS) and buffer, respectively

On the day of infection, cells were harvested by centrifugation at 800 × *g* for 10 min and washed in minimal medium (PYG without glucose, yeast extract, or tryptone for *A. castellanii* and PYNFH medium lacking FBS and buffer for *V. vermamoeba*). The cell suspension was then adjusted to a final concentration of 5 × 10⁵ cells/ml. Amoeba suspensions (1 ml) were seeded into 24-well cell culture plates. Infectious bacteria were collected from freshly grown colonies on CYE agar plates and resuspended in Fraquil medium to an optical density of OD₆₀₀ = 0.1 (~1 × 10⁸ CFU/ml). Infections were initiated at a multiplicity of infection (MOI) of 1 by adding 5 µl of the bacterial suspension to each well. Initial colony forming units (CFU) counts were determined from the gently mixed supernatant immediately after infection. Infected cultures were incubated at 37°C for 96 h, with bacterial growth monitored by CFU enumeration at 24-h intervals.

## Results

### *Legionella anisa* isolates recovery and genomic characterization

Among the isolates obtained, all four *L. anisa* isolates (Table 1) were recovered from a single location: a bath faucet with an internal hot–cold water mixing valve. Recovery required a pretreatment or enrichment step for three of the four isolates: two were recovered from amoebal coculture combined with acid treatment, and one from acid treatment alone; only one isolate was recovered by direct plating without pretreatment (Table 1). The *L. anisa* isolates were further subjected to whole-genome sequencing (WGS), and assembly quality is summarized in Table 1. Short and long reads were combined to generate closed genomes. Each genome consists of a single chromosome averaging 4.5 Mbp, along with multiple plasmids of varying sizes (Table 1). Functional annotation with Prokka identified an average of 4351 coding sequences, 9 rRNA genes, and 43 tRNA genes per genome (Table S3). The assemblies showed low contamination (1.9%) and 100% completeness, as determined by MiGA.

**Table 1:** Summary of assembly characteristics of new *L. anisa* isolates from a HCF in Rimouski, QC Canada in 2024.

Isolate name	Isolation plate	Assembly type	Contig type	Length (bp)	Depth (x)
SPF1048	*T. pyriformis* amplification + acid treatment + GVPC	Hybrid	Chromosome	4 559 261	1.00
			Plasmid (pLA170A)	170 213	1.52
			Plasmid (pLA54)	54 850	1.08
SPF1049	*T. pyriformis* amplification + acid treatement + GVPC	Hybrid	Chromosome	4 547 368	1.00
			Plasmid (pLA170B)	170 213	1.55
			Plasmid (pLA51A)	51 349	1.47
			Plasmid (pLA12)	12 369	1.87
SPF1050	GVPC	Hybrid	Chromosome	4 580 502	1.00
			Plasmid (pLA170C)	170 213	1.62
			Plasmid (pLA51B)	51 349	1.37
SPF1051	Acid treatment + CYE	Hybrid	Chromosome	4 562 996	1.00
			Plasmid (pLA170D)	170 213	1.62
			Plasmid (pLA51C)	51 349	1.37

* Plasmids sharing the same base name (e.g. pLA170) are identical in sequence across isolates. Letter suffixes (A–D) denote isolate-specific presence.

### Phylogenetic analysis and recombination detection

Phylogenetic analysis was carried out to compare the isolates from this study (*n* = 4) with each other and with all publicly available *L. anisa* (*n* = 76) genomes. Most publicly available isolates originated from the USA (*n* = 53), with a smaller number from Europe (*n* = 3). Information on isolation source was largely missing from the NCBI database (*n* = 53), except for a few reported as environmental (e.g. dental unit or tap water). Details of the publicly available genomes are provided in Table S1.

**Figure 1:**
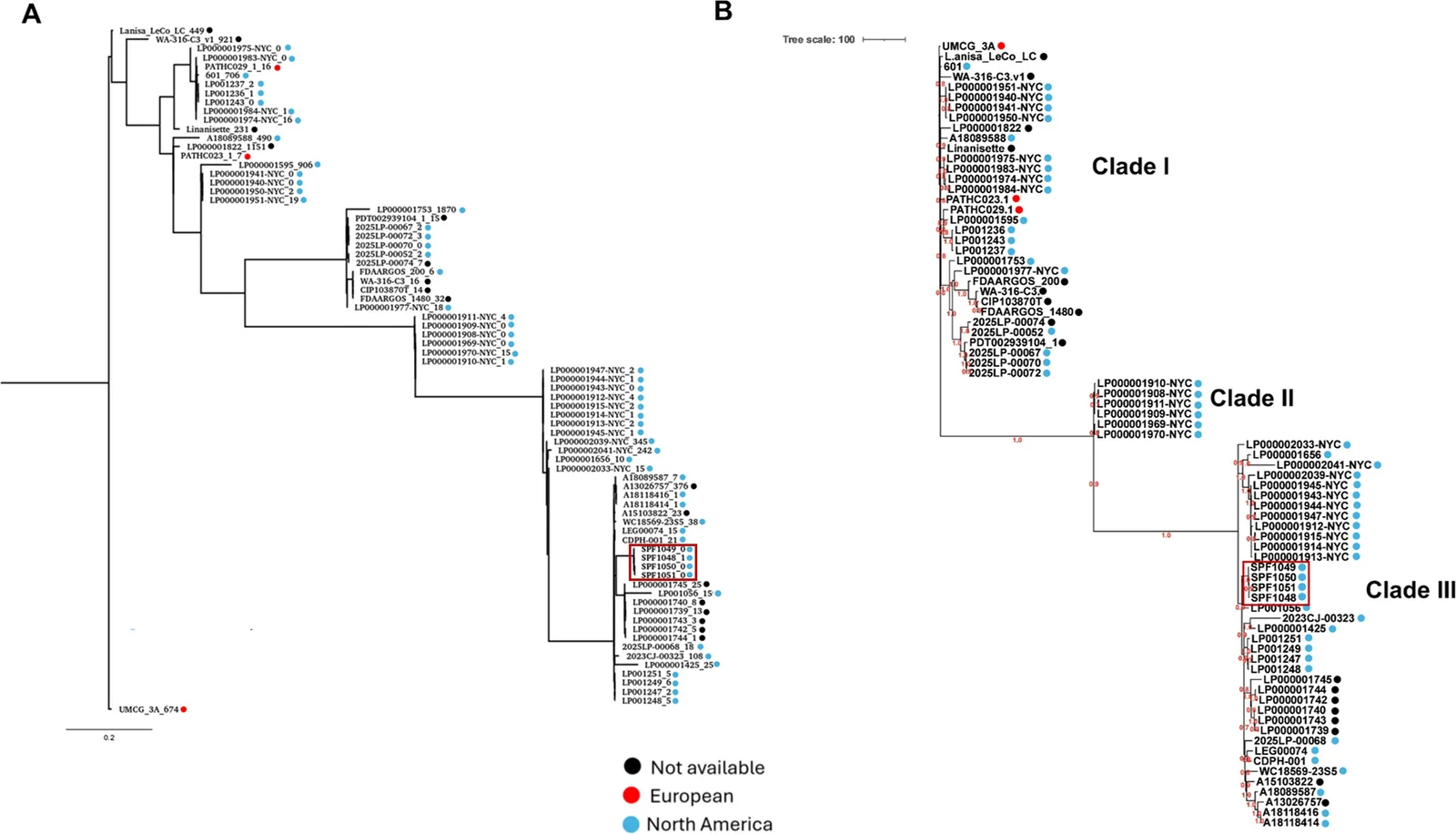
Phylogenetic analysis of *L. anisa* isolates. (A) Maximum-likelihood phylogeny of all *L. anisa* genomes based on SNPs from both core and accessory regions. Isolates are annotated with colored circles corresponding to their isolation source (when available). The tree is rooted at *L. anisa* isolate UMCG_3A. The numbers following the taxa name denotes number of SNP unique to the isolate. The scale bar indicates the number of nucleotide substitutions per site. (B) Phylogeny of four *L. anisa* genomes sequenced in this study and 73 reference genomes from the NCBI database. A maximum-likelihood tree was constructed using SNPs falling outside recombination regions, identified with Gubbins, with the –tree-builder option set to IQ-TREE. Branch support was assessed using 1000 bootstrap replicates, and transfer bootstrap support was enabled (–transfer-bootstrap: TRUE) to provide more reliable support values for phylogenetic branches. In the final tree, visualized using iTOL, only bootstrap values greater than 70% (0.7) are displayed. The scale bar indicates the number of SNPs per site. The source of isolates is indicated when available. The *L. anisa* genomes sequenced in this study are highlighted with a rectangle.

We first applied kSNP4 analysis to provide a broad view of genetic relatedness of the genomes, as this method incorporates all SNPs across the genomes. Further, to complement this, we used Gubbins to reconstruct a tree based only on vertically inherited SNPs by masking putative recombinant regions, thereby minimizing the impact of horizontal gene transfer on phylogenetic inference. The kSNP analysis showed a broad population structure with several distinct lineages (Fig. 1A). Although sharp phylogenetic resolution was not evident, likely due to the inclusion of all SNPs both within and outside recombination regions, the isolates sequenced in this study (SPF1048, SPF1049, SPF1050, and SPF1051) formed a tight monophyletic cluster, differing by maximum 1 SNP among each other. These isolates grouped closely with CDPH-001, a clinical isolate collected from California, USA, and several other environmental isolates collected from USA between 2018 and 2025.

**Figure 2:**
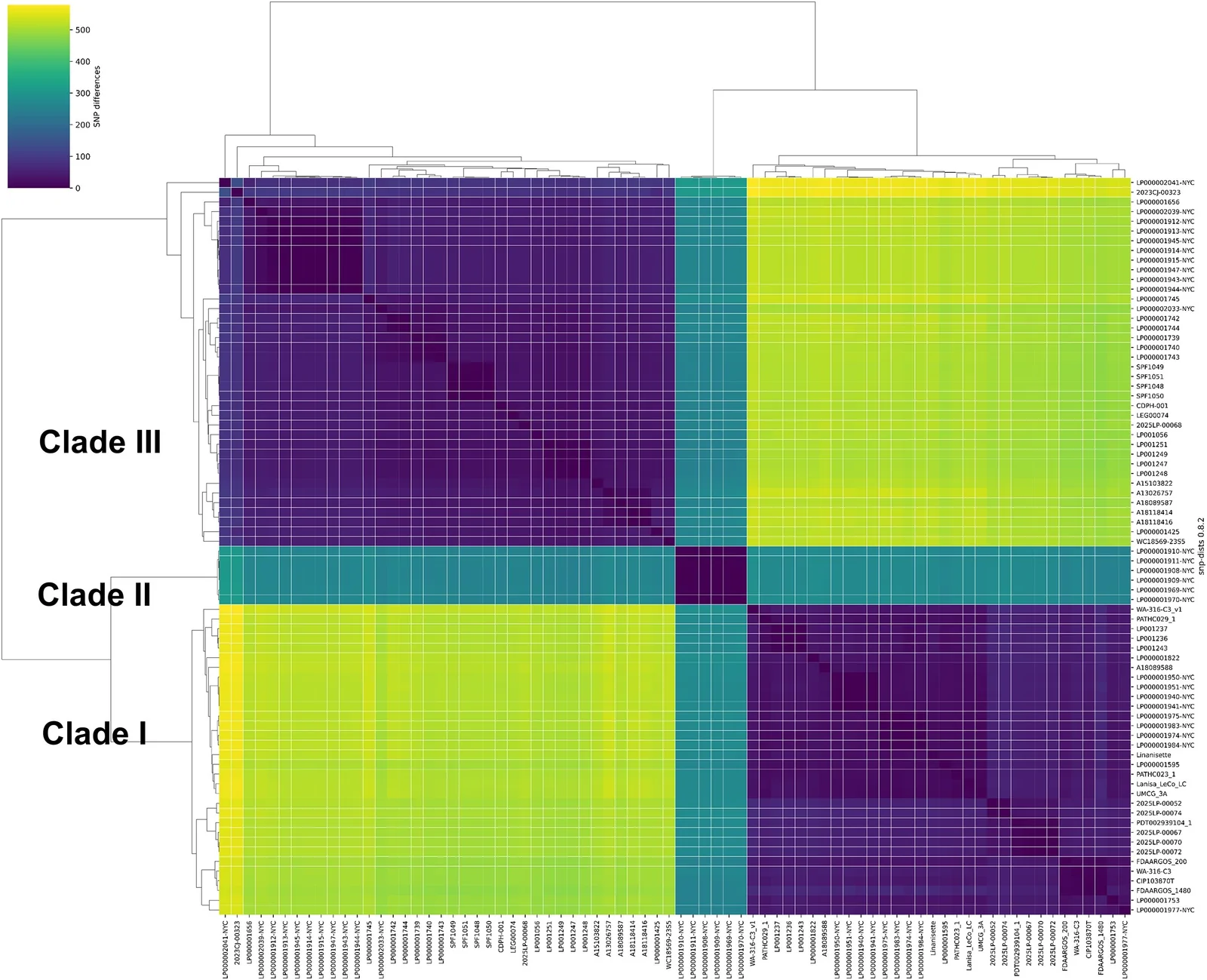
Clustered heatmap showing pairwise SNP (outside the recombination regions) distance between *L. anisa* genomes from this study and NCBI reference genomes.

Figure 1(B) shows the maximum-likelihood tree generated from SNPs outside recombinant regions. Three distinct clusters were observed in the SNP-based phylogeny, designated clade I (33 isolates), clade II (6 isolates), and clade III (38 isolates). This clustering did not strongly correspond to geographical origin. Furthermore, the SNP-distance matrix confirmed the presence of three well-separated clades (Fig. 2). Within each clade, pairwise SNP differences were generally low (<100), whereas divergence between clades I and III was ~500 SNPs, indicating substantial genomic differentiation. The four isolates sequenced in this study clustered tightly within clade III, differing by a maximum of one pairwise SNPs, consistent with a clonal relationship.

Since Gubbins also performs recombination detection, it identifies SNPs located within recombinant regions, quantifies recombination events, and calculates the r/m ratio to estimate the relative contribution of recombination to sequence diversity (Croucher et al. 2015). We examined how recombination shaped genome diversity and how many recombination events were detected. Table S4 summarizes SNPs within recombinant regions, the number of recombination events, and their contribution to diversity for individual genomes, while Fig. S1 shows the phylogenetic distribution of these events.

Across the dataset, most genomes (*n* = 61) showed no detectable recent recombination relative to the reference genome (r/m = 0), indicating that their recent diversification occurred through clonal SNP accumulation. Nevertheless, elevated r/m values (30–70) were observed within clades II and III, reflecting the presence of shared recombinant regions in these lineages. These recombinant segments were not introduced recently in the sampled genomes but were acquired earlier and subsequently inherited during clonal expansion. Importantly, removal of recombinant regions did not alter the overall population structure, as the core phylogeny continued to resolve three distinct clades. (Table S4, Fig. S1).

### *Legionella anisa* harbors several plasmids

**Figure 3:**
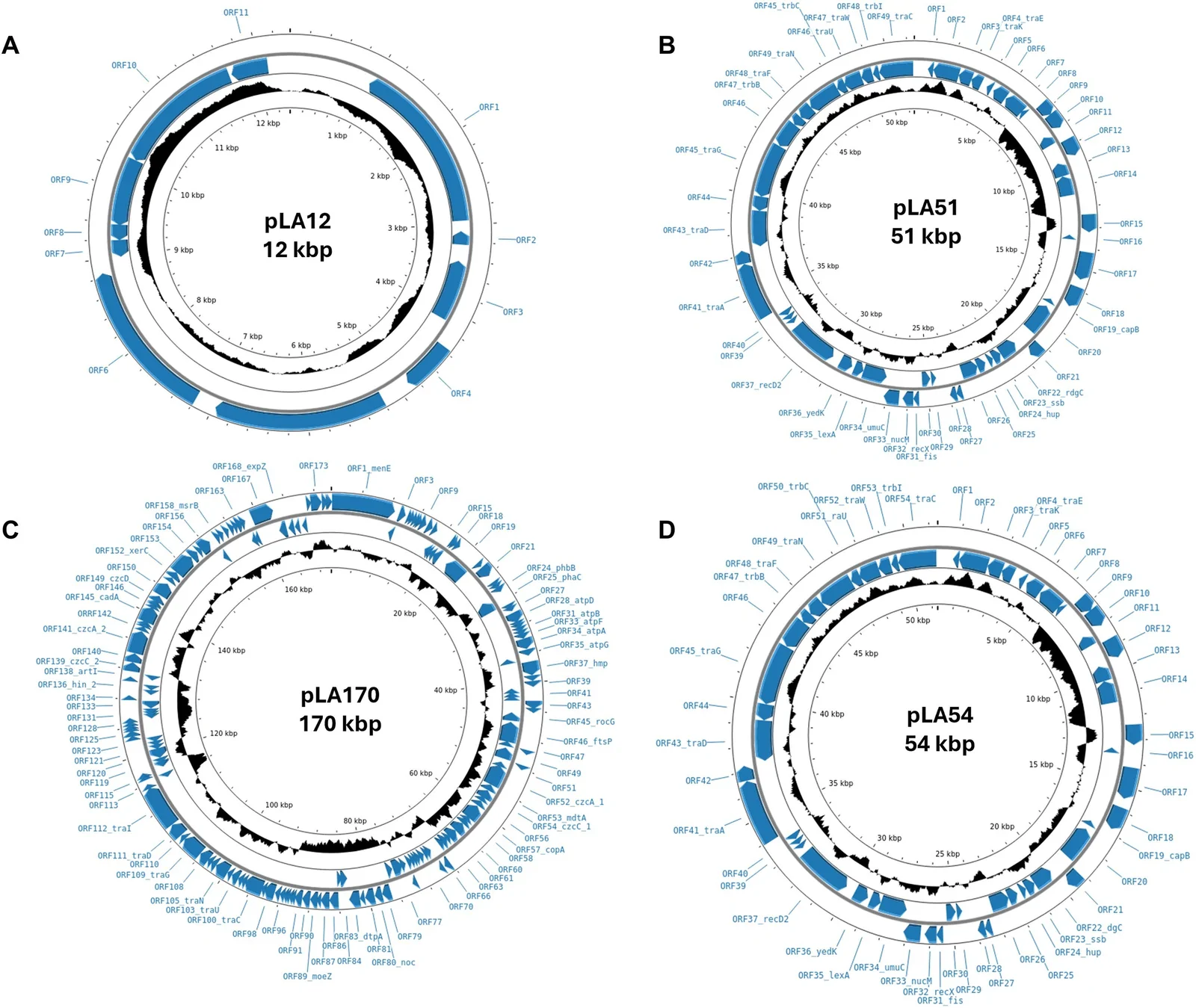
Circular maps of plasmids identified in *L. anisa* isolates. (A) pLA12, (B) pLA51, (C) pLA170, and (D) pLA54. Outer rings show coding sequences annotated by Prokka, with predicted gene functions indicated. Inner plots display GC content.

After hybrid assembly, varying numbers of circular contigs were identified in all *L. anisa* isolates sequenced and characterized in this study. Excluding the largest contig in each isolate, which represented the chromosome based on size and coverage, the remaining contigs were considered putative plasmids (Table 1, Fig. 3). Because some plasmids were detected in multiple isolates, we performed similarity analysis with BLAST, which showed that plasmid pLA170 was present in all isolates with 100% sequence similarity. Likewise, pLA51 was detected in all isolates with 100% similarity. Following visual confirmation as smaller, high-coverage contigs in Bandage, further validation was performed using MOB-suite, which identifies hallmark plasmid functions, bins plasmid contigs, and assigns plasmids to reference clusters based on Mash similarity, together with predicted replicon and mobility types. Detailed plasmid typing results are provided in Table S5. According to this analysis, pLA12 showed the closest match to plasmid CP031654 (plasmid pCARB35_01) from *Escherichia coli* strain UK_Dog_Liverpool. Plasmids pLA51 and pLA54 showed the closest match to CP015957, an unnamed plasmid from *L. pneumophila* strain E9_O, while pLA170 showed the closest match to an unnamed plasmid from *L. pneumophila* strain E2_N. Plasmid pLA12 was predicted to be non-mobilizable, whereas the remaining plasmids were predicted to be conjugative. All plasmids were further annotated with Prokka using their closest plasmid sequences as references (Table S6). pLA12, the smallest plasmid, contained only 11 coding sequences, most with unknown function. pLA51 and pLA54 contained 50 and 54 coding sequences, respectively, with most genes encoding plasmid conjugative transfer proteins. pLA170 contained 176 coding sequences, including genes for conjugative transfer proteins, ATP synthase subunits, tyrosine recombinase, copper-exporting P-type ATPases, the metal cation efflux protein CzcD, 2-succinylbenzoate–CoA ligase, and numerous hypothetical proteins. No AMR or virulence genes were detected in any plasmid when screened with ABRicate against the CARD and ResFinder databases.

### Comparative pangenome analysis

**Figure 4:**
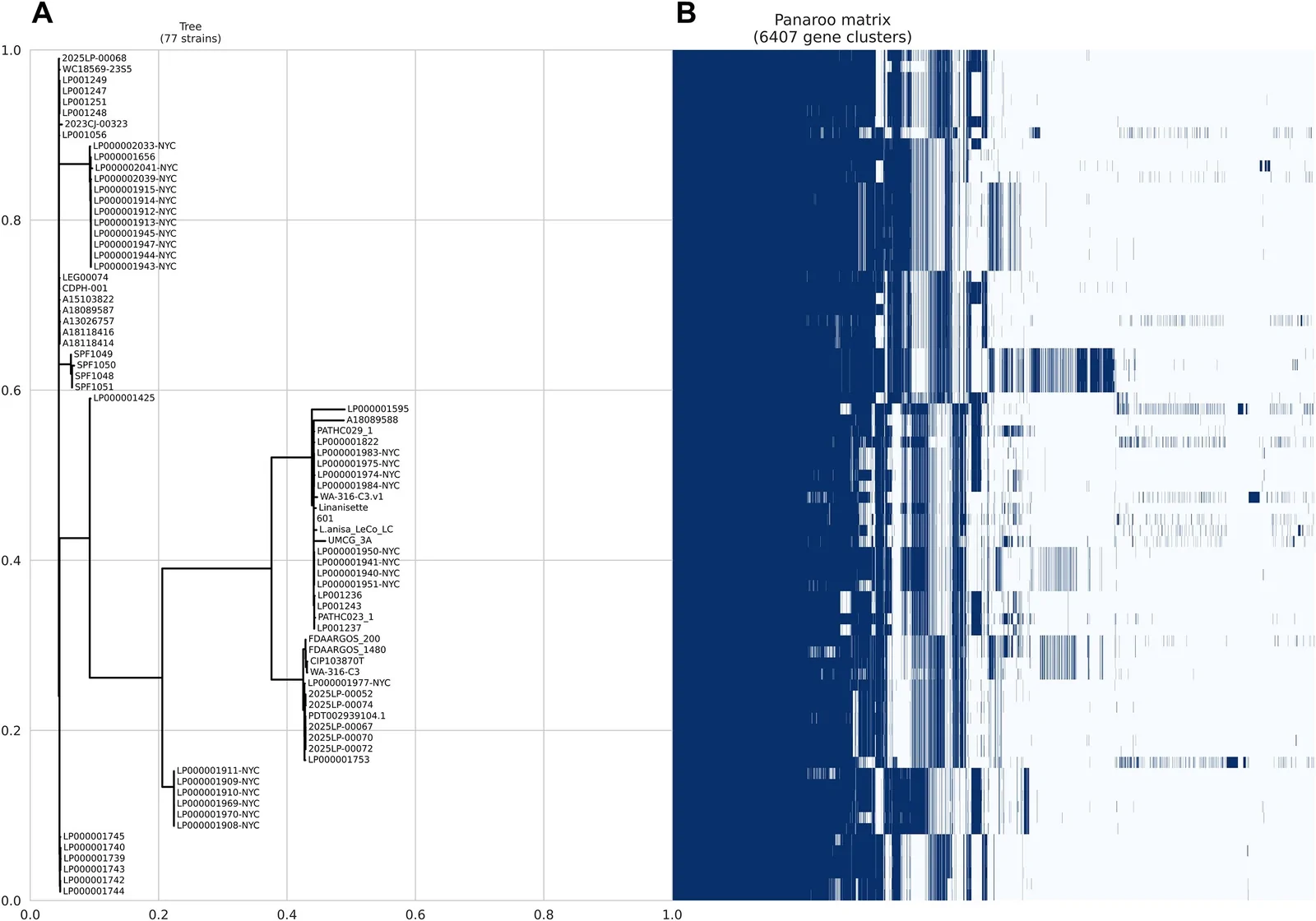
Pangenome analysis of *L. anisa* isolates. (A) Phylogenetic tree constructed based on core genes. (B) Pangenome matrix illustrating gene presence (blue) and absence (white).

Comparative pangenome analysis showed that all the genomes shared 3032 core genes. Out of a total of 6407 genes, the accessory genome comprised ∼50% (*n* = 3375) of the total pangenome, of which 58% (*n* = 2079) cloud genes, 35% (*n* = 1074) shell genes, and 6% (*n* = 222) soft core genes (Fig. 4). The *L. anisa* isolates from this study showed the same close clustering based on gene presence/absence with only about 20 accessory genes, most of them encoding hypothetical proteins. Detailed pangenome analysis of all genomes is given in Table S7.

### Detection of AMR, and virulence genes

Abricate analysis performed on all four isolates sequenced in this study against the card database Three genes named FEZ-1 beta-lactamase, OXA-29, and LpeB were identified in all the isolates. Presence of virulence genes in all *L. anisa* genomes was also evaluated with Abricate against VFDB analysis using % COVERAGE and %IDENTITY set at 60%. *Legionella pneumophila* subsp. pneumophila str. Philadelphia 1 was used as the reference. Majority of VF identified were common in all the genomes (Detail given in File S8) except six genes with variable presence including *sodC, lpg1661, lpg0405, lem23, legS2*, and *ddhA* (Fig. S2). These are all known or putative *L. pneumophila* virulence-associated genes, many linked to intracellular survival, modulation of host processes, or secretion systems.

### Infection and proliferation of *L. anisa* in amoeba host cells

**Figure 5:**
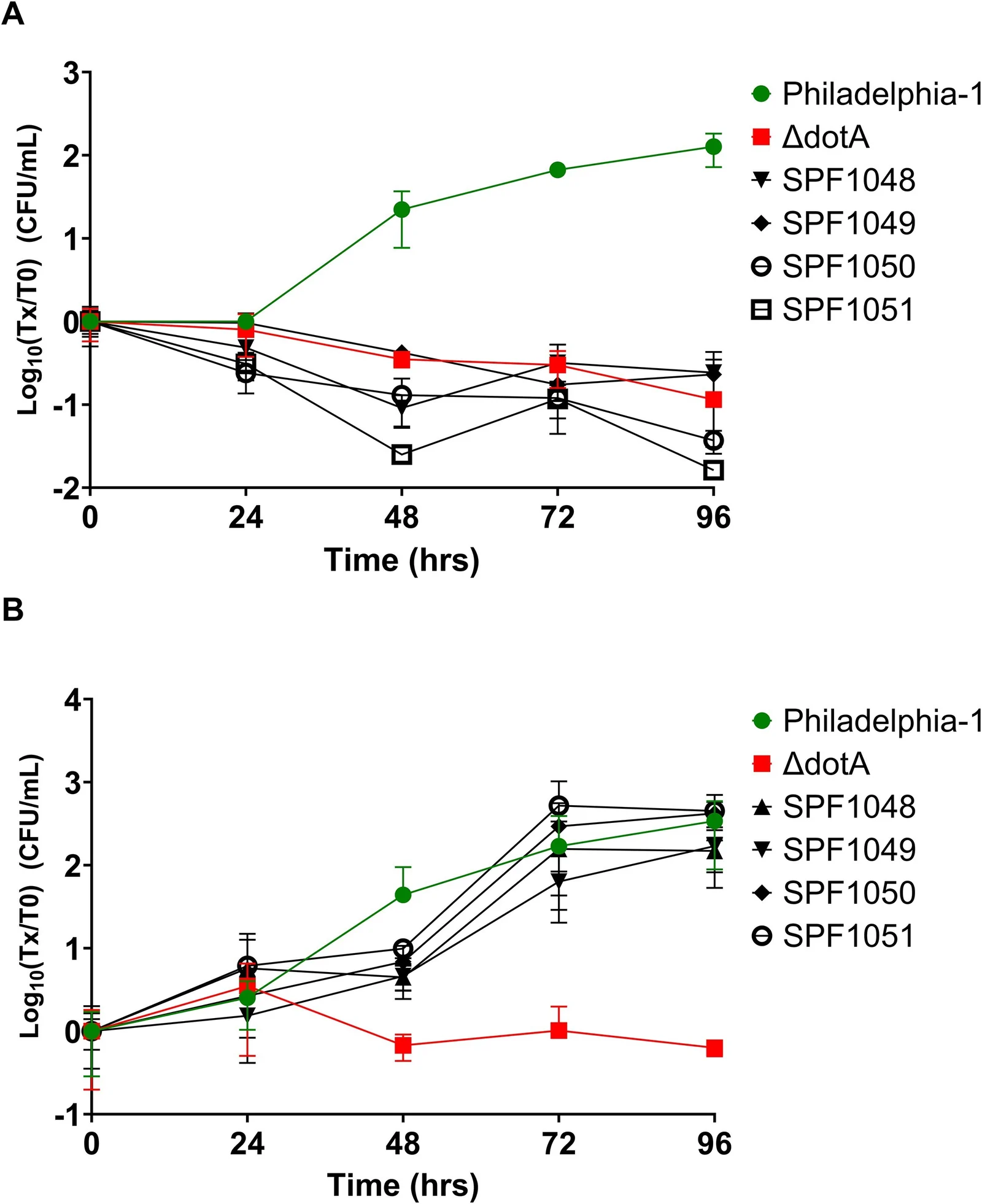
Intracellular proliferation of *L. anisa* strains from this study (*n* = 4) in *V. vermiformis* (A) and *A. castellanii* (B) at 37°C. The *L. pneumophila* strain Philadelphia-1 was used as a positive control, while the Δ*dotA* mutant served as a negative control. Host cells were infected at an MOI of 1, and bacterial growth was monitored over 96 h by sampling every 24 h and plating on CYE agar for CFU enumeration. Data represent the mean ± standard deviation of three biological replicates.

The ability of *L. anisa* strains isolated in this study (*n* = 4) to infect and replicate within protozoan hosts was assessed using *A. castellanii* and *V. vermiformis* at 37°C (Fig. 5). In *A. castellanii*, all *L. anisa* strains exhibited increase in intracellular bacterial counts over time, indicating intracellular proliferation. Growth was comparable to *L. pneumophila* Philadelphia-1 positive control, with bacterial count increasing steadily over the course of the experiment. In *V. vermiformis*, none of the *L. anisa* strains showed a significant increase in CFU counts over the 96-h infection period. Bacterial numbers remained stable or decreased slightly in comparison to *L. pneumophila* Philadelphia-1. The infection-negative dotA strain was unable to grow in both amoeba, as expected.

## Discussion

*Legionella anisa* has been frequently detected in EWS and has been associated with human infections. However, there are few studies focusing on its genomic diversity and population structure compared to *L. pneumophila*. Although numerous *L. anisa* genomes (draft or complete) are now publicly available, most have not been analysed collectively with respect to phylogeny and accessory genome variation, factors that may influence environmental persistence and pathogenic potential. The goal of this study was to provide complete genomic characterization for four new environmental *L. anisa* isolates collected from Rimouski, Canada, and to compare them with the broader collection of available genomes using phylogenomic, recombination, and pangenome approaches.

At a broader scale, both kSNP- and Gubbins-based phylogenies revealed similar grouping of *L. anisa* genomes. However, kSNP, which includes both vertically inherited and recombination-associated SNPs, showed less distinct clustering between lineages, whereas Gubbins, which excludes recombinant regions and reflects vertical inheritance, revealed tighter clade structure. Notably, isolates from this study displayed the same close clustering in both analyses, indicating strong phylogenetic relatedness regardless of the inclusion of recombination. The four isolates sequenced in this study clustered tightly and differed by no more than one SNP, consistent with clonal expansion from a recent common ancestor and a shared environmental source. While no lineage-specific recombination was detected in these isolates, their placement within clade III indicates inheritance of recombination events that occurred ancestrally and contributed to the diversification of this lineage.

At a broader scale, the SNP-distance matrix further confirmed the presence of three well-separated clades, characterized by low pairwise SNP distances within clades (<100 SNPs) and markedly higher divergence between clades, with ~500 SNPs separating clades I and III. While there is no universally defined SNP threshold for delineating clades in *L. anisa*, the magnitude and structure of these distances are consistent with patterns reported in whole-genome sequencing studies of other *Legionella* species (Garner et al. 2019). A recent *L. anisa* WGS study based on wgMLST similarly reported very low allelic distances among closely related isolates, contrasted by substantially higher allelic divergence when compared to other publicly available *L. anisa* genomes, supporting the existence of distinct genomic lineages (Fleres et al. 2018).

Moreover, with reference to population genomics studies of *L. pneumophila*, where core-genome SNP analyses have been extensively applied, it could be seen that epidemiologically linked or closely related isolates differ by only a small number of SNPs, whereas unrelated isolates and distinct phylogenetic lineages are separated by hundreds to thousands of SNPs. Within this framework, the ∼500 SNP divergence observed between *L. anisa* clades in this study is indicative of substantial genomic differentiation rather than within-lineage variation. Taken together, the concordant phylogenetic signals obtained using kSNP and Gubbins, the pronounced SNP divergence between clades support the presence of genetically distinct *L. anisa* lineages and highlight the role of recombination in shaping long-term population structure.

However, it was difficult to determine whether any of the broader clustering patterns reflected region-specific lineages because essential metadata such as sampling origin, source, and date of collection were missing for many isolates. Improved and standardized metadata reporting would greatly enhance the interpretation of phylogenetic analyses, allow better evaluation of potential geographic clustering, and strengthen the utility of *L. anisa* genomes for future infection investigations.

Comparative pangenome analysis demonstrated that *L. anisa* strains share ~47% of their genes as core genome, reflecting moderate genomic conservation. The accessory genome showed substantial variation, consistent with the dynamic genome characteristic of *Legionella* species, driven by mobile genetic elements such as prophages, plasmids, and transposons (Gomez-Valero and Buchrieser 2013, Fleres et al. 2018). In our isolates, multiple plasmids of various sizes were identified. Some plasmids displayed high sequence similarity to those found in *E. coli* and *L. pneumophila*, suggesting possible intergenera and interspecies horizontal gene transfer, as previously reported (Gomez-Valero et al. 2011). These plasmids carried genes associated with conjugation, heavy-metal resistance and other stress-resistance related genes. We previously showed that a plasmid carried by a clinical isolate of *L. pneumophila*, collected from a mixed infection conferred increased copper resistance (Najeeb et al. 2025). Given the recognized role of plasmid-encoded functions in bacterial adaptation and persistence under stress (Zwanzig 2021, Cameron and Faucher 2025), it is possible that the plasmids identified in this study may have potential persistence related roles. Further investigation into their functional relevance would therefore be valuable.

Regarding AMR genes, three were identified across all genomes including FEZ-1 β-lactamase, OXA-29, and LpeB. These genes were also reported in previous studies (Massip et al. 2017, Svetlicic et al. 2023). The presence of these AMR genes is unlikely to be clinically significant since β-lactams are not used for legionellosis treatment (Tewolde et al. 2025). No AMR genes were detected on the plasmids.

Virulence gene screening showed that *L. anisa* genomes harbor a range of known *Legionella* virulence factors, although a few (e.g. *sodC, lpg1661, lpg0405, lem23, legS2*, and *ddhA*) were absent in our isolates. Many of these genes encode components or effectors of the Dot/Icm type IV secretion system or enzymes that protect against host oxidative stress and modulate eukaryotic signaling. The absence of these *L. pneumophila* associated virulence genes in our *L. anisa* isolates may suggest host specificity, however, this claim would need future experimental validation since these virulence genes have been found to exhibit redundancy regarding their function (Best and Abu Kwaik 2018).

Intracellular replication of *L. anisa* within protozoan host cells has previously been reported, although outcomes vary depending on the host species and experimental conditions. Early work by Fields and colleagues demonstrated that *L. anisa* could infect and multiply within cultures of *Hartmannella vermiformis* (*Vermamoeba vermiformis*), while failing to infect guinea pigs or replicate within *T. pyriformis* or human mononuclear cells (Fields et al. 1990). These findings suggested that some *Legionella* species may exhibit a restricted intracellular host range, particularly among protozoa (Fields et al. 1990). The *L. anisa* isolated in this study were capable of efficient intracellular replication in *A. castellanii* at 37°C, reaching levels comparable to those observed for *L. pneumophila* Philadelphia-1. These findings are consistent with those reported by Croze et al. (2021), who observed robust intracellular replication of the *L. anisa* strain 1025 in *A. castellanii* at 37°C. Notably, the study also reported marked strain-dependent variability, as another *L. anisa* strain (ATCC 35291), isolated from a sink faucet, failed to replicate in any of the amoebal hosts tested (Croze et al. 2021). Together, these observations highlight pronounced heterogeneity in intracellular behavior among *L. anisa* strains. In contrast to our observations in *A. castellanii*, none of the *L. anisa* isolates tested here showed detectable intracellular replication in *V. vermiformis*. While this differs from the results reported previously (Fields et al. 1990), such discrepancies are likely attributable to differences in bacterial strain background, protozoan host strains, or experimental conditions. Overall, our findings support the concept that *L. anisa* exhibits host-dependent intracellular behavior and suggest that successful replication may be restricted to specific protozoan hosts. However, future studies examining a wider range of protozoan and mammalian host cells will be important to further clarify the ecological and clinical significance of these host-specific interactions.

In conclusion, by employing a hybrid assembly approach, we generated four complete, high-quality genomes of *L. anisa* isolated from water systems in Rimouski, Canada. Comparative genomic analyses placed these isolates within a clonal *L. anisa* lineage, while broader phylogenetic and recombination analyses highlighted the lineage-specific contribution of recombination to genetic diversity across the species. The detection of multiple plasmids with diverse genetic content further underscores the potential of *L. anisa* for gene exchange and adaptation in aquatic environments. Moreover, infection assays demonstrated that these isolates are capable of efficient intracellular replication in *A. castellanii* at 37°C, while exhibiting host-dependent limitations in *V. vermiformis*, emphasizing the importance of protozoan host specificity in shaping *L. anisa* persistence. Together, these findings expand the current genomic framework for *L. anisa* and provide a foundation for future studies aimed at elucidating the genomic and ecological factors underlying its environmental persistence and potential pathogenicity.

## Supplementary Material

fnag100_Supplemental_Files

## Data Availability

Assembled genomes and raw reads are available from GenBank under project number PRJEB107406.
