# Supplementary material for: Comparative genomic analysis reveals distinct population structure in Legionella anisa
Source: FEMS Microbiol Lett. 2026 Sep 3;373:fnag100. doi: 10.1093/femsle/fnag100 (PMC13591635; doi:10.1093/femsle/fnag100)
Supplement: Supplementary file 1 [file fnag100_supplemental_files.zip › Supplementary_Figure_Legends.docx]

**Supplementary Figure 1:** Recombination detection with Gubbins. The left panel shows a maximum-likelihood phylogeny of all analyzed *L. anisa* genomes, with three major clades (Clade I–III) indicated. The right panel displays recombination events detected by Gubbins across the same genomes. Each row corresponds to a genome, and each column represents a genomic position where recombination was inferred. Red blocks indicate recombinant regions, with intensity reflecting the number of inferred recombination events, as shown in the color scale (0–10). Genomes with little or no recombination show mostly white space, whereas highly recombinant genomes display dense red patterns.

**Alt text:** Phylogenetic tree paired with a recombination matrix, where each row is a genome and each column a genomic position; red blocks mark inferred recombination events, showing Clade III genomes with dense, widespread red patterns and Clade I genomes with sparser recombination concentrated in fewer columns.

**Supplementary Figure 2:** Presence/absence of variable virulence genes across *L. anisa* genomes. Heatmap showing the distribution of selected virulence-associated genes (rows) across all *L. anisa* genomes analyzed (columns). Dark blue indicates gene presence, while white indicates absence. Several virulence genes, including *icmE/dotG*, *lem23*, *lem8*, and *lpg1661*, exhibited variable presence across isolates, whereas others, such as *sodC* and *ddhA*, were widely conserved.

**Alt text:** Heatmap of variable virulence genes across *L. anisa* genomes, dark blue for present and white for absent.
