# Supplementary material for: Comparative genomic analysis reveals distinct population structure in Legionella anisa
Source: FEMS Microbiol Lett. 2026 Sep 3;373:fnag100. doi: 10.1093/femsle/fnag100 (PMC13591635; doi:10.1093/femsle/fnag100)
Supplement: Supplementary file 1 [file fnag100_supplemental_files.zip › Table_S2_Supplementry_Data.docx]

**Table S2: Sequencing characteristics of short- and long-read data for isolates from this study**

| **Isolate** | **Sequencing technology** | **Number of Reads** | **Mean read length** | **High-quality bases** | **Read length N50 (bp)** | **Total bases** |
| --- | --- | --- | --- | --- | --- | --- |
| **SPF1048** | Illumina | 1.968954 M | 287 | \| 535.745123 M (Q20; 94.74%) | - | 565.449114 M |
|  | Oxford Nanopore | 102,165 | 2533.7 | 117.9 M (Q15; 33.1%) | 6368.0 | 258,855,612.0 M |
| **SPF1049** | Illumina | \| 7.73793 M | 288 | 2.113434 G  (Q20; 94.52) | - | \| 2.235750 G |
|  | Oxford Nanopore | 90,294.0 | 2,572.5 | 28204 (Q15; 31.2%) | 2,572.5 | 232,282,613.0 M |
| **SPF1050** | Illumina | 2.304254 M | 287 | 625.339327 M (Q20; 94.40) | - | 662.372858 M |
|  | Oxford Nanopore | 101,245.0 | 3,144.6 | 32893 (Q15; 32.5%) | 7,806.0 | 318,372,457.0 M |
| **SPF1051** | Illumina | 3.36818 M | 287 | 921.767702 M (Q20; 95.04) | - | \| 969.772876 M |
|  | Oxford Nanopore | 72,773.0 | 1,923.8 | 21093 (Q15; 29.0%) | 5,395.0 | 140,003,182.0 M |
