# Supplementary material for: Comparative genomic analysis reveals distinct population structure in Legionella anisa
Source: FEMS Microbiol Lett. 2026 Sep 3;373:fnag100. doi: 10.1093/femsle/fnag100 (PMC13591635; doi:10.1093/femsle/fnag100)
Supplement: Supplementary file 1 [file fnag100_supplemental_files.zip › Table_S5_Supplementry_Data.docx]

| **Plasmid** | **Size (bp)** | **Mob-suite cluster** | **Predicted mobility*** | **Relaxase type** | **MPF type** | **Closest reference plasmid (accession)** | **Reference host** | **Mash neighbor identification** | **Mash distance*** |
| --- | --- | --- | --- | --- | --- | --- | --- | --- | --- |
| pLA12 | 12,369 | Singleton | Non-mobilizable | - | - | - |  | *Escherichia coli* | - |
| pLA51 | 51,349 | Rep_cluster_866 | Conjugative | MOBQ | MPF_F | CP015957 | *L. pneumophila* | NC_009966_00026 | 0.059 |
| pLA54 | 54,850 | Rep_cluster_866 | Conjugative | MOBQ | MPF_F | CP015957 | *L. pneumophila* | NC_009966_00026 | 0.061 |
| pLA170 | 170,213 | Singleton | Conjugative | MOBF | MPF_F | CP015948 | *L. pneumophila* | NC_018141_00143 | 0.081 |

**Table S4: MOB-suite-based typing of plasmids detected in *Legionella anisa* isolates included in this study**

***Mash distance** reflects k-mer–based similarity and does not represent nucleotide percent identity.

*Predicted mobility categories were assigned by MOB-suite based on detected mobility genes.

“–” indicates feature not detected.
